# TGF-β1 induces the formation of vascular-like structures in embryoid bodies derived from human embryonic stem cells

**DOI:** 10.3892/etm.2014.1721

**Published:** 2014-05-19

**Authors:** YAN WANG, DE-JIAN QIAN, WEN-YU ZHONG, JUN-HONG LU, XIANG-KAI GUO, YI-LIN CAO, JU LIU

**Affiliations:** 1 Department of Plastic Surgery, Provincial Qianfoshan Hospital Affiliated to Shandong University, Jinan, Shandong 250014, P.R. China; 2 Department of Gynecology and Obstetrics, Jinan Central Hospital, Jinan, Shandong 250013, P.R. China; 3 Department of Plastic and Reconstructive Surgery, Shanghai Ninth People’s Hospital, Shanghai 200011, P.R. China; 4 Medical Research Center, Shandong Provincial Qianfoshan Hospital, Jinan, Shandong 250014, P.R. China

**Keywords:** human embryonic stem cell, transforming growth factor-β, embryoid body, endothelial cell

## Abstract

Human embryonic stem cells (ESCs) can differentiate into endothelial cells in response to stimuli from extracellular cytokines. Transforming growth factor (TGF)-β1 signaling is involved in stem cell renewal and vascular development. Previously, human ESCs were isolated from inner cell mass and a stable ESC line was developed. In the present study, the effects of extracellular TGF-β1 were investigated on human ESC-derived embryoid bodies (EB) in suspension. The structures of the EBs were analyzed with light and electron microscopy, while the cellular composition of the EBs was examined via the expression levels of specific markers. Vascular-like tubular structures and cardiomyocyte-like beating cells were observed in the EBs at day 3 and 8, respectively. The frequencies of vascular-like structures and beating cells in the TGF-β1 treated group were significantly higher compared with the control group (84.31 vs. 12.77%; P<0.001; 37.25 vs. 8.51%; P<0.001, respectively). Electron microscopy revealed the presence of lumens and gap junctions in the sections of the tubular structures. Semiquantitative polymerase chain reaction revealed elevated expression levels of CD31 and fetal liver kinase-1 in EBs cultured with TGF-β1. In addition, extensive staining of von Willebrand factor was observed in the vascular-like structures of TGF-β1-treated EBs. Therefore, the results of the present study may aid the understanding of the underlying mechanisms of human ESC differentiation and improve the methods of propagating specific cell types for the clinical therapy of cardiovascular diseases.

## Introduction

Human embryonic stem cells (ESCs) are self-renewing pluripotent cells that can differentiate into a wide variety of cell types under specific *in vitro* culture conditions (1). These cells offer a promising source to treat a number of human diseases by providing an unlimited supply of different cell types, including endothelial cells (ECs) for therapeutic neovascularization and cardiomyocytes for repairing heart failure damage (2–5). Although human ESC-derived ECs and cardiomyocytes have been reported, the underlying mechanisms of differentiation and maintenance of cell fate remain unclear. Understanding the molecular biology of human ESCs may help to identify the factors promoting a cardiovascular fate and improve ESC culture conditions, which may lead to novel therapeutic methods for the treatment of cardiovascular diseases.

Several cytokines have been shown to facilitate the differentiation of ESCs into ECs. One such cytokine is transforming growth factor (TGF)-β1, a highly conserved protein produced by a variety of cells (6). TGF-β1 is a member of the TGF-β superfamily, which plays a key role in the regulation of human ESCs (6,7). TGF-β1 activates its receptors through ligand binding, which is followed by oligomerization of serine/threonine receptor kinases and the phosphorylation of the cytoplasmic signaling SMAD proteins that regulate the transcription of targeted genes (8). Targeted gene deletions of TGF-β1 and its receptors, TGF-βR1/2*,* result in abnormal vascular development of mouse embryos, particularly of the yolk sac, leading to embryonic lethality (9–11). A number of studies have commonly indicated a requirement for TGF-β1 to support self-renewing cultures of human ESCs. For example, the inhibition of TGF receptors with a compound resulted in rapid differentiation of human ESCs (12–14).

When allowed to differentiate in suspension, ESCs develop cystic embryoid bodies (EBs) that have specific features of early post-implantation embryos. Notably, EB formation from mouse ESCs has already been extensively utilized and validated as an *in vitro* model of early mouse development (15–18). Mouse ESCs transfected with a 1.7 kb cDNA of porcine TGF-β1 were shown to be able to differentiate into EBs with outspread tubular structures and increased endothelial marker expression (19). However, human and mouse ESCs have disparate responses to extracellular stimuli (7). In a study of human ESCs collected from the yolk sac, Poon *et al* found that TGF-β1 inhibited the expression of endodermal and hematopoietic markers, which is in contrast to the observations with mouse ESCs (7). To date, the roles of TGF-β1 in human ESCs derived from inner cell mass (ICM) remain unclear.

In previous research, human ESCs were isolated from the ICM of human blastocysts and were shown to have a normal karyotype, express specific pluripotent markers and propagate for extended periods of time (20). In the present study, these human ESCs were cultured, differentiated into EBs in suspension culture and transferred into medium containing TGF-β1. The structures of the EBs were examined by light and electron microscopy, while the cellular composition of these structures was analyzed via the expression levels of several endothelial-specific markers.

## Materials and methods

### Cell culture

A human ESC line was previously established (21), with approval from the Internal Review Board on Human Subjects Research and Ethics Committees at Shanghai Ninth People’s Hospital (Shanghai, China). Briefly, human ESCs were isolated from day 6 blastocysts and transferred onto a feeder layer of mitomycin-C- (Sigma-Aldrich, St. Louis, MO, USA) inactivated mouse embryonic fibroblasts (MEFs) in human ESC culture medium. The medium consisted of Dulbecco’s Modified Eagle’s medium (DMEM; Invitrogen Life Technologies, Carlsbad, CA, USA) supplemented with 20% KnockOut Serum Replacement (Invitrogen Life Technologies), 1% non-essential amino acids, 1 mM L-glutamine, 0.1 mM β-mercaptoethanol and 4 ng/ml basic fibroblast growth factor (bFGF; R&D Systems, Minneapolis, MN. USA). Cells were incubated at 37°C with 5% CO_2_, and colonies were passed every 5–7 days using 1 mg/ml IV collagenase.

### Induction and formation of EBs

To induce EB formation, human ESC colonies were dissociated into small cell mass mechanically and grown in dishes covered with 1% agar in ESC culture medium containing 1×10^9^ mol/l retinoic acid (R2625; Sigma-Aldrich). Following culturing for 3–5 days, the cells aggregated and formed EBs. The EBs were then transferred into dishes covered with 0.1% gelatin and cultured in serum-free DMEM containing 3 ng/ml TGF-β1 (240-B-010; R&D Systems) or as controls with serum-free DMEM only. The culture medium was changed every 24 h, and the morphological changes of the EBs were examined daily under a phase-contrast microscope. The images of beating cells of the EBs were stored on a videotape using a Nikon CCD camera (Nikon Corporation, Tokyo, Japan).

### Semiquantitative polymerase chain reaction (PCR) analysis

Total RNA isolation from the EBs was performed using TRIzol reagent (Invitrogen Life Technologies), according to the manufacturer’s instructions. An aliquot of 2 μg total RNA from each sample was used for the synthesis of cDNA using a High-Capacity cDNA Reverse Transcription kit (Applied Biosystems, Inc., Foster City, CA, USA). The first-strand cDNA (equivalent of 40 ng reverse-transcribed RNA) was amplified in a final volume of 20 μl with 1 unit *Taq*DNA polymerase (Invitrogen Life Technologies) and 10-pmol samples of each primer. The oligonucleotide primers are listed on Table I. The thermal-cycle program was as follows: 95°C for 5 min (one cycle); 94°C for 1 min, 58°C for 1 min and 72°C for 1 min (30 cycles); and 72°C for 5 min (one cycle). To ensure the accuracy of the quantitative results, the number of PCR cycles for each set of primers was validated in the linear range of amplification, and all cDNA samples were adjusted to yield equal amplifications of β-actin, which was used as the internal control. The PCR products were visualized by ethidium bromide staining following 1.2% agarose gel electrophoresis.

### Immunofluorescence staining

EBs were fixed in 4% paraformaldehyde for 10 min, washed with phosphate-buffered saline (PBS) containing 0.25% Triton and incubated with rabbit anti-von Willebrand factor (vWF) antibodies (Dako, Ely, UK) at a working concentration of 1:100 for 1 h at 37°C. Following washing with PBS, the EBs were incubated with goat anti-rabbit IgG (H&L) secondary antibodies conjugated with fluorescein (Abnova Corporation, Taipei City, Taiwan) at a working concentration of 1:50 for 45 min at 37°C. The samples were then mounted using glycerol and photographed with a fluorescent microscope. The negative control was prepared using the same protocol without primary antibody incubation.

### Electron microscopy

Observations with a scanning electron microscope (SEM) were firstly conducted. The EBs were fixed with 2% glutaric dialdehyde, rehydrated in PBS, fixed again with osmic acid and washed in PBS. Next, the EBs were dehydrated by gradient alcohol with amyl acetate, dried with a Critical Point Dryer, sputter-coated by an ionic sprayer meter and observed with a SEM (S450; Hitachi, Ltd., Tokyo, Japan). Observations were also performed with a transmission electron microscope (TEM). The EBs were fixed with 2% glutaric dialdehyde for 1 h and osmic acid for 1 h, dehydrated by gradient alcohol and incubated with 1:1 acetone embedding liquid in infiltration. The EBs were embedded with EPON and cut into ultra-thin sections, which were then observed with a TEM (JEM-1200EX; Jeol Ltd., Tokyo, Japan).

## Results

### Formation of EBs with tubular structures from human ESCs stimulated by TGF-β1

**Figure 1:**
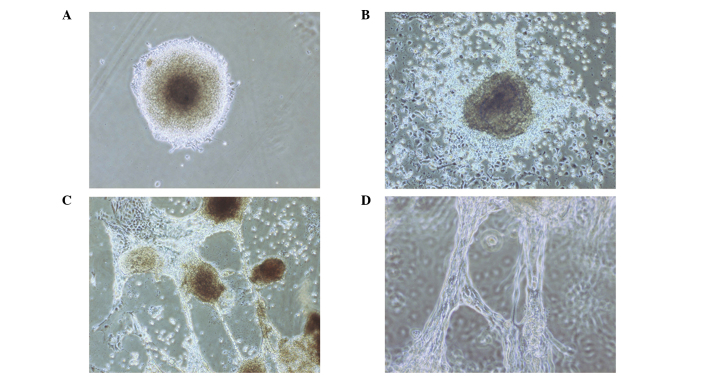
Morphology of human ESC-derived EBs (unstained images). (A) EBs in the control group exhibited a smooth margin; (B) EBs treated with TGF-β1 demonstrated vascular-like structures with epithelial-like and round cells (magnification, ×20), and (C) tube-like structures connected to the EBs (magnification, ×40); (D) tube-like structures were observed at a higher magnification (magnification, ×100). TGF, transforming growth factor; EBs, embryoid bodies; ESC, embryonic stem cell.

**Figure 2:**
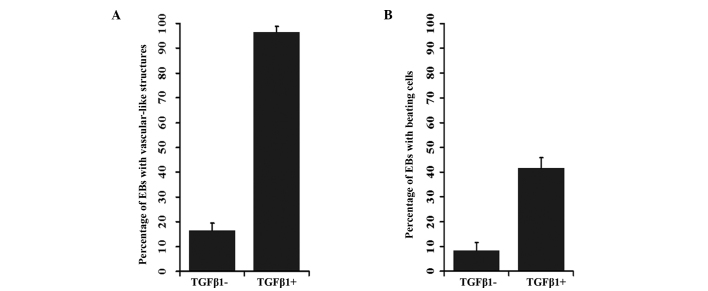
TGF-β1 induced vascular and cardiac-like structures in human ESC-derived EBs. EBs were developed in suspension with and without TGF-β1. Percentage of EBs with (A) vascular-like structures (n=3; P<0.001) and (B) beating cells (n=3; P<0.001). TGF, transforming growth factor; EBs, embryoid bodies; ESC, embryonic stem cells.

Colonies of human ESCs were dissociated into small cell mass and grown on 1% agar. Following culturing in media containing retinoic acid for 4 days, the cell mass formed EBs, which adhered to the dishes and became semispheres. The EBs were then cultured in serum-free DMEM containing 3 ng/ml TGF-β1, and epithelial-like and round cells appeared around the EBs after 1 day. On day 3, tube-like structures deriving from the round cells were observed, while the cells gradually transformed from round to flat shapes (Fig. 1A). Tube-like structures radiated from EBs to the outside (Fig. 1B). These tubular structures connected, extended and joined with each other to form a network (Fig. 1C and D). The frequencies of these structures in the control and TGF-β1 treated groups were 12.77 and 84.31%, respectively (P<0.001; Fig. 2A; Table II). At day 8, cardiomyocyte-like beating cells were observed in the EBs that had been treated with TGF-β1. In addition, at day 10, beating cells were observed in 37.25% of the EBs treated with TGF-β1, while only in 8.51% of the EBs in the control (P<0.001; Fig. 2B, Table III).

### Characterization of the vascular-like structures by electron microscopy

**Figure 3:**
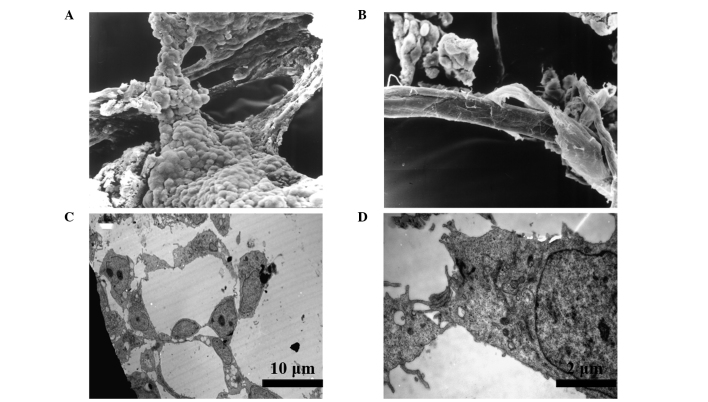
Vascular-like structures of EBs were observed with electron microscopy. SEM images revealed that (A) the tube wall was composed of round and flat cells, and (B) with the elongation of tube-like structures, the tube wall became smooth. TEM images revealed that (C) vascular-like structures had lumens surrounded by flat endothelial-like cells and (D) neighboring cells were connected by tight junctions. SEM, scanning electron microscope; TEM, transmission electron microscope; EBs, embryoid bodies.

SEM images exhibited a three-dimensional morphology of the EBs. The tubular structures were composed of round and flat cells, which were morphologically similar to ECs (Fig. 3A). These structures joined with each other and constituted a network of tubes with a smooth surface (Fig. 3B). Further observations using a TEM revealed the presence of lumens and gap junctions in the section of tubular structures (Fig. 3C). The lumens were surrounded by several round and flat cells, which were connected by tight junctions (Fig. 3D). These structures were similar to those of capillaries during early embryo development.

### Examination of the endothelial-like cells in the EBs derived from human ESCs

**Figure 4:**
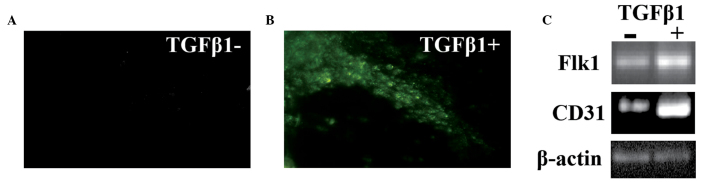
Expression of endothelial cell specific markers on EBs with vascular-like structures. Immunostaining of vWF on vascular-like structures of EBs in the (A) control and (B) TGF-β1-treated groups. (C) Semiquantitative PCR revealed increased expression levels of FLK1 and CD31 in the EBs treated with TGF-β1. TGF, transforming growth factor; EB, embryoid bodies; vWF, von Willebrand factor; PCR, polymerase chain reaction; FLK1, fetal liver kinase-1.

Expression levels of vWF, an established endothelial marker (22), were analyzed on the EBs by immunofluorescence staining. On the tubular structures of the EBs stimulated by TGF-β1, marked vWF staining was observed, while only a limited number of vWF positive cells were observed on the tubular structures from the control group (Fig. 4A and B). In addition, the EBs were harvested and total RNA was extracted for semiquantitative PCR analysis. The two markers of endothelial cells, CD31 and FLK1, exhibited higher expression levels in the EBs treated with TGF-β1 compared with those from the control group (Fig. 4C), indicating that TGF-β1 induced an increase in differentiated cells with endothelial cell characteristics.

## Discussion

Previously, a human ESC line derived from ICM was established and characterized. In the current study, these ESCs were employed and the development of EBs stimulated by TGF-β1 was investigated. Light and electron microscopic observations demonstrated that TGF-β1 induced the *in vitro* differentiation of embryonic stem cells into ECs, as well as the formation of vascular-like structures. The vascular identity of the cells in the EBs was validated by the expression of endothelial cell markers. In addition, TGF-β1 promoted the differentiation of cardiomyocyte-like beating cells in EBs.

Human HSCs require coculture on mitotically inactivated MEFs, which cannot be substituted by the addition of leukemia inhibitory factor (1,23). However, the addition of TGF-β1 and other soluble factors, including bFGF and insulin-like growth factor, demonstrated a supportive role in the maintenance and propagation of human ESCs in culture (12,14,24). In addition, TGF-β1 has been shown to affect the cell fate decisions during epithelial-mesenchymal transition by upregulating surviving-associated proteins (25). In this study, TGF-β1 was shown to differentially regulate the differentiation of human ESCs and promote an endothelial cell fate. Although the mechanisms are not clear, upregulation of FLK1 expression may play an important role in this process. The FLK1 gene encodes vascular endothelial growth factor receptor 2 (VEGFR2), the major functional receptor of VEGF in ECs (26). Upon stimulation by TGF-β1, SMAD3 becomes phosphorylated and forms a complex with SMAD4. These SMAD complexes translocate into the nucleus, where they bind to the promoter region of the FLK1 gene, thereby activating transcription (27). In the current study, EBs stimulated by TGF-β1 exhibited marked mRNA expression of FLK1, supporting the role in mediating TGF-β1-regulated cell fate decisions.

TGF-β1 and its isoforms regulate a variety of diverse biological functions, and the role of TGF-βl in vascular development is complicated. TGF-β1 stimulates *in vivo* angiogenesis (28–30), but directly inhibits the proliferation of ECs *in vitro* (31–34). TGF-β1-stimulated induction of angiogenesis requires EC apoptosis, which occurs via autocrine/paracrine stimulation of VEGF expression and signaling via VEGFR2 (35). *In vitro*, TGF-β1 induces tube formation when ECs are cultured inside three-dimensional collagen gels (36,37). In the current study, TGF-β1 was shown to promote vascular tube formation in EBs. Tube formation is a key process during vascular development, and vascular endothelial cadherin (VE-cadherin) is the cell adhesion molecule essential for this process (38,39). VE-cadherin facilitates homotypic interactions between ECs and is strictly required for the polarization of ECs (40,41). TGF-β1 induces the rearrangement of the adherens junction complex by separating FLK1 from VE-cadherin and increasing the associations between β-catenin and FLK1 or VE-cadherin (42). Therefore, TGF-β1 may promote cells with an endothelial identity to interact with each other and form tubular-like structures in EBs.

A previous study demonstrated that transfection of ESCs with the porcine TGF-β1 gene permits vascular development from murine ESCs in culture (19). Under electron microscopy, the tubular structures observed in transfected murine EBs were similar to those from the human EBs stimulated by extracellular TGF-β1 in the present study. All these structures were composed by the cells expressing EC markers. Therefore, extracellular TGF-β1 and the transfected TGF-β1 gene can induce vascular-like structures in EBs, possibly by activating downstream TGF-β1 signaling in ESCs. The results indicated that TGF-β1 is closely associated with the formation of vascular structure.

Cardiomyocyte-like cells derived from human ESCs have been actively pursued as a novel therapeutic to repair regions of damaged hearts. Studies on mouse embryonic development identified the TGF-β superfamily member, activin A, and bone morphogenic protein 4, as key inducers of mesoderm and cardiovascular differentiation (43). In addition, TGF-β1 induces the differentiation of human cardiac progenitor cells into beating cardiomyocytes with characteristic cross striations (44). In the present study, TGF-β1 was shown to promote the differentiation of human ESCs to cardiomyocyte-like beating cells in EBs, consistent with the role of TGF-β1 in supporting stem cell differentiation towards functional cardiomyocytes. Therefore, components of the TGF-β signaling pathway may be used to manipulate human ESCs to regenerate myocardium for cell-based therapy.

In conclusion, the present study has demonstrated that extracellular TGF-β1 promotes a cardiovascular fate of human ESCs in culture. The results indicate that TGF-β1 can be used as a stimulator for the production of human ESC-derived ECs, cardiomyocytes and forming vascular tube structures. These observations may help to improve the methods of propagating specific cell types for the clinical therapy of cardiovascular diseases. Further studies are required to dissect the molecular mechanisms underlying the function of TGF-β1 on cell fate decisions and vascular tube formation.

## Figures and Tables

**Table I:** Oligonucleotide primers used for semiquantitative PCR analysis.

Gene	Primer sequence	Annealing temperature, °C	Cycles, n	Size, bp^a^
FLK1	5′-CCTACCCCACACATTACATGG-3′ 5′-TTTTCCTGGGCACCTTCTATT-3′	58	30	200
CD31	5′-AGGAAAGCCAAGGCCAGG-3′ 5′-CCTTGCTGTCTAAGTCCT-3′	58	30	354
β-actin	5′-AGGTGACAGCATTGCTTCTG-3′ 5′-GCTGCCTCAACACCTCAAC-3′	58	30	188

a Size of the amplified cDNA product in base pairs

FLK1, fetal liver kinase-1; PCR, polymerase chain reaction.

**Table II:** Number of EBs with vascular-like structures in the control and TGF-β1-treated groups.

	Control		TGF-β1	
		
Group	EBs formed, n	EBs with VS, n (%)	EBs formed, n	EBs with VS, n (%)
1	15	2 (13.33)	18	15 (83.33)
2	16	3 (18.75)	13	13 (100)
3	16	1 (6.25)	20	15 (75)
Total	47	6 (12.77)	51	43 (84.31)

VS, vascular-like structures; EB, embryoid body; TGF, transforming growth factor. Groups 1–3 are repeats of the same experiment.

**Table III:** Number of EBs with beating cells in the control and TGF-β1-treated groups.

	Control		TGF-β1	
		
Group	EBs formed, n	EBs with beating cells, n (%)	EBs formed, n	EBs with beating cells, n (%)
1	15	2 (13.33)	18	6 (33.33)
2	16	1 (6.25)	13	4 (30.77)
3	16	1(6.25)	20	9 (45)
Total	47	4 (8.51)	51	19 (37.25)

EB, embryoid body; TGF, transforming growth factor.
